# Physical activity and associations with health-related quality of life in adults born small for gestational age at term: a prospective cohort study

**DOI:** 10.1186/s12887-023-04256-y

**Published:** 2023-08-28

**Authors:** Cathrin Vano Mehl, Silje Dahl Benum, Kristina Anna Djupvik Aakvik, Atle Kongsvold, Paul Jarle Mork, Eero Kajantie, Kari Anne I. Evensen

**Affiliations:** 1 Department of Clinical and Molecular Medicine, Norwegian University of Science and Technology, NTNU, Trondheim, N-7491 Norway; 2 Department of Public Health and Nursing, Norwegian University of Science and Technology, Trondheim, Norway; 3 Finnish Institute for Health and Welfare, Public Health Promotion Unit, Helsinki and Oulu, Finland; 4 Clinical Medicine Research Unit, MRC Oulu, Oulu University Hospital and University of Oulu, Oulu, Finland; 5 Children’s Hospital, Helsinki University Hospital and University of Helsinki, Helsinki, Finland; 6 Unit for Physiotherapy Services, Trondheim Municipality, Trondheim, Norway; 7 Children’s Clinic, St. Olavs Hospital, Trondheim University Hospital, Trondheim, Norway; 8 Department of Rehabilitation Science and Health Technology, Oslo Metropolitan University, Oslo, Norway

**Keywords:** Objective measure, Self report, SF-36, Health status, Long-term outcome, Adult, Longitudinal studies, Human activities, Sedentary behavior, Wearable electronic devices

## Abstract

**Background:**

Adults born small for gestational age (SGA) have increased risk of adverse health outcomes. Physical activity (PA) is a key determinant of health and health-related quality of life (HRQoL). We aimed to investigate if being born SGA at term is associated with lower objectively measured and self-reported PA during adulthood. We also examined if objectively measured and self-reported PA were associated with HRQoL.

**Methods:**

As part of the 32-year follow-up in the NTNU Low Birth Weight in a Lifetime Perspective study, SGA and non-SGA control participants wore two tri-axial accelerometers for seven days (37 SGA, 43 control), and completed the International Physical Activity Questionnaire (IPAQ) (42 SGA, 49 control) and the Short Form 36 Health Survey (SF-36) (55 SGA, 67 control). Group differences in objectively measured daily metabolic equivalent of task (MET) minutes spent sedentary (lying, sitting), on feet (standing, walking, running, cycling), on the move (walking, running, cycling) and running/cycling, and group differences in self-reported daily MET minutes spent walking and in moderate and vigorous PA were examined using linear regression. Associations with SF-36 were explored in a general linear model.

**Results:**

Mean (SD) daily MET minutes on the move were 218 (127) in the SGA group and 227 (113) in the control group. There were no group differences in objectively measured and self-reported PA or associations with HRQoL. In the SGA group, one MET minute higher objectively measured time on the move was associated with 4.0 (95% CI: 0.6–6.5, *p* = 0.009) points higher SF-36 physical component summary.

**Conclusion:**

We found no differences in objectively measured and self-reported PA or associations with HRQoL between term-born SGA and non-SGA control participants in adulthood.

**Supplementary Information:**

The online version contains supplementary material available at 10.1186/s12887-023-04256-y.

## Background

Being born small for gestational age (SGA; birth weight < 10^th^ percentile for gestational age) is associated with various health risks throughout life [1]. These include unfavorable adult body composition [2], increased cardiometabolic risk factors [3] and increased psychiatric morbidity [4]. Additionally, low birth weight at term has been associated with lower cardiorespiratory fitness [5] and lower grip strength [6], which could contribute to or be a consequence of lower physical activity (PA). A recent review indicates that most studies using self-reports found an association between low birth weight and increased sedentary time, while most studies using objective measurements found no association [7]. Using self-reports along with objective measurements may provide complementary information about habitual PA. To date there are no studies of objectively measured PA in adults born SGA at term.

The World Health Organization (WHO) guidelines emphasize the importance of regular PA and a reduction in sedentary time to maintain or improve health [8]. Adults are recommended to do at least 150–300 min of moderate PA (MPA); or at least 75–150 min of vigorous PA (VPA) per week; or an equivalent combination of moderate and vigorous PA (MVPA) [8]. Regular PA has a broad range of health benefits, prevents noncommunicable diseases and reduces symptoms of anxiety and depression, amongst others [9]. Furthermore, studies have found a positive association between PA and health-related quality of life (HRQoL) [10, 11]. The association was consistent across different measures of PA, although the association with HRQoL was stronger for objective measures [10]. In the NTNU Low Birth Weight in a Lifetime Perspective (NTNU LBW Life) study, we found that the physical HRQoL of adults born SGA at term on average deteriorated from 20 to 28 years of age, and then remained stable until 32 years, compared with the control group of non-SGA adults [12].

Since PA seems to be associated with HRQoL, and HRQoL decreased among young adults born SGA, it is important to gain more knowledge about their PA and whether PA is associated with HRQoL. We therefore aimed to investigate if being born SGA at term is associated with lower objectively measured PA and lower self-reported PA during adulthood. We also investigated if objectively measured and self-reported PA were associated with HRQoL.

## Methods

### Study design and study population

As part of the NTNU LBW Life study, two groups of adults born at term (gestational age ≥ 37 weeks) in 1986–1988 have been followed prospectively from birth until 32 years of age. The SGA group had a birth weight < 10^th^ percentile and the non-SGA control group had a birth weight ≥ 10^th^ percentile. The participants have attended several study visits over the years.

The participants were initially included as part of a multicenter study that recruited pregnant women before 20 weeks of gestation [13, 14]. They were eligible if they were carrying a singleton and had been pregnant once or twice before. A total of 1249 women in the region of Trondheim, Norway, consented to participate. A 10% random sample (*n* = 132) was selected for follow-up using a sealed envelope method, representative of the pregnant population at the study site. Women at high risk of delivering an SGA infant were selected for follow-up if they fulfilled one or more defined risk criteria; a previous child with low birth weight (LBW) or perinatal death, pre-pregnancy weight < 50 kg, cigarette smoking at conception or chronic diseases, i.e., hypertension, renal or heart disease (*n* = 390). Women in the random sample and in the high-risk group were followed through pregnancy and their babies were examined at birth. The remaining women were not selected for detailed follow-up (*n* = 727). All infants born SGA at term to mothers in either of the three groups were included in the SGA group (*n* = 104). The control group (*n* = 120) comprised all infants born non-SGA from the random sample only.

**Fig. 1:**
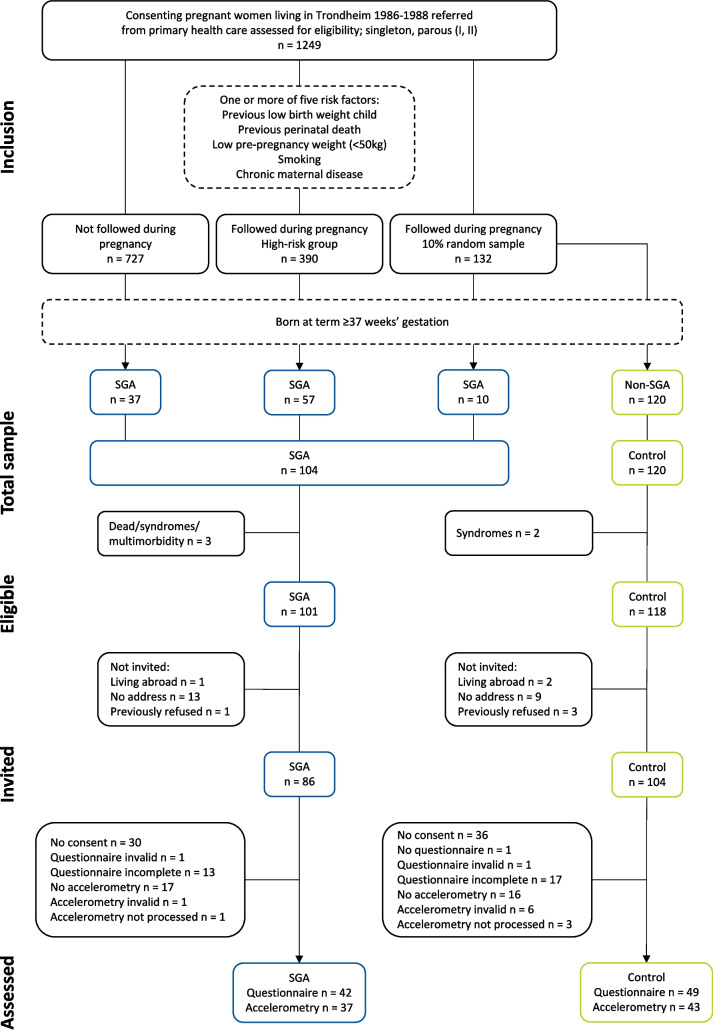
Flow of participants at 32 years. SGA = small for gestational age

At the 32-year follow-up, data were collected from September 2019 through March 2021. PA was examined as part of a larger data collection, including anthropometric measurements, examination of lung function, physical fitness, motor, and visual function. Individuals who were unable to meet for clinical examination were invited to answer questionnaires. Flow of the participants at the 32-year follow-up is visualized in Fig. 1.

#### SGA participants

The SGA group included 104 individuals born at term with a birth weight < 10^th^ percentile for gestational age, corrected for sex and parity, according to a reference standard using data from the Norwegian Medical Birth Registry [13]. Gestational age was determined based on the first day of the mother’s last menstrual period (LMP) when this was recalled accurately ± 3 days. An ultrasound estimate was used if the LMP-based gestational age was not recalled or differed by more than 14 days from the ultrasound-based gestational age. Individuals with congenital syndromes, multimorbidity, or who died before follow-up were excluded (*n* = 3). At the 32-year follow-up, 15 individuals born SGA were living abroad, could not be reached, or had previously refused to participate. Out of 86 invited SGA adults, 30 did not consent, leaving 56 SGA participants (65% of 86 invited and 55% of 101 eligible). Of these, 42 (19 men, 23 women) SGA participants completed the self-report PA questionnaire and 37 (17 men, 20 women) SGA participants had valid accelerometer data (Fig. 1).

#### Control participants

The control group included 120 individuals born at term with a birth weight ≥ 10^th^ percentile. Two individuals with congenital syndromes were excluded. At the 32-year follow-up, 14 control adults were living abroad, could not be reached, or had previously refused to participate. Out of 104 invited subjects, 36 did not consent, leaving 68 control participants (65% of 104 invited and 58% of 118 eligible). Of these, 49 (22 men, 27 women) control participants completed the self-report PA questionnaire and 43 (20 men, 23 women) control participants had valid accelerometer data (Fig. 1).

#### Non-participants

There were no substantial differences between participants and those who did not consent to participate at the 32-year follow-up regarding gestational age, birth weight, birth length, head circumference, ponderal index, maternal age at delivery, parental socioeconomic status (SES) or sex in either group [12].

#### Background characteristics

At birth, gestational age, birth weight, birth length, head circumference, sex and maternal age were recorded. Ponderal index (g/cm^3^) was calculated based on birth weight and length.

At the 14- and 19-year follow-ups, data on the parents’ education and occupation were collected. Parental SES was calculated according to Hollingshead’s Two Factor Index of Social Position [15]. The social class was rated from 1 (lowest) to 5 (highest).

At the 32-year follow up, adult height and weight were measured, and used to calculate the participants’ body mass index (BMI, kg/m^2^). Height was measured to the nearest 0.1 cm. Weight was measured by bioelectric impedance analysis to the nearest 0.1 kg, using a Seca medical Body Composition Analyzer (Seca® mBCA 515).

### Objectively measured physical activity

#### Data collection and data processing

Physical activity was measured by two tri-axial accelerometers (AX3, Axivity, Newcastle, UK), positioned centrally on the lower back at the third lumbar segment and on the front of the right thigh approximately 10 cm above the upper border of the patella. To attach the accelerometers, a 5 × 7 cm moisture permeable film (Opsite Flexifix; Smith & Nephew, Watford, United Kingdom) was attached to the skin. The accelerometer was then positioned on top of the film using double-sided tape and covered with a second film layer of 10 × 8 cm. The accelerometers were configured to record continuously for seven days at 50 Hz with a range of ± 8 g, using the OmGui software (version 1.0.0.37; Open Movement, Newcastle University, United Kingdom). The raw data were stored on a 512 MB internal memory. After completing the recording, participants returned the accelerometers in a pre-stamped envelope. Raw CWA data were downloaded using the OmGui software, visually inspected for content of data and converted to CSV format where the signals from the two sensors were synchronized. A machine learning model was used to classify the raw accelerometer data into six key PA types and postures, i.e., lying down, sitting, standing, walking, running, and cycling. The machine learning model has been validated, showing an overall accuracy of 96% for detecting the six different behaviors in a free-living setting [16, 17]. A separate machine learning model was used to classify no-wear time.

The machine learning model classified one of the six activities for each five second window of data. To remove noise, a majority voting was performed for each minute (i.e., 12 five second windows), and the most frequent activity was then classified, resulting in one observation per minute. The first and last day of monitoring were excluded, and if no-wear time was classified for over one hour, data from that day on were excluded. Therefore, only days with 24 h of valid accelerometer data were included in the analyses. Total time spent in each PA type and posture during the whole monitoring period was calculated. Daily average minutes spent in each PA type and posture were calculated by dividing the total time by the total days of monitoring. Weekly averages were calculated by multiplying the daily averages by seven. Daily averages based on data from weekdays only and weekend days only were calculated. Moreover, participants were only included in the analyses if they met the minimum required days of monitoring, which were at least one weekday (Monday through Friday) and one weekend day (Saturday or Sunday) of monitoring. In total, 17 SGA and 16 control participants did not wear accelerometers, while one SGA and six control participants did not meet the minimum required days of monitoring. For one SGA and three control participants who wore accelerometers, data were not processed successfully.

#### Outcome variables

The volume of activity was quantified by metabolic equivalent of task (MET) minutes, computed by multiplying the estimated MET value of each posture and PA type by the minutes spent in each posture and PA type per day [18]. The MET values for each of the postures and PA types used in calculating MET minutes were as follows: lying down = 1.0; sitting = 1.3; standing = 1.8; walking = 2.8; running = 7.0; cycling = 4.0 [18].

The six PA types and postures were used to create four PA categories: 1) sedentary (i.e., the sum of time spent lying down and sitting); 2) on feet (i.e., the sum of time spent standing, walking, running and cycling); 3) on the move (i.e., the sum of time spent walking, running, and cycling); and 4) running/cycling (i.e., the sum of time spent running and cycling).

We examined the proportion of participants fulfilling the cut-offs derived from the WHO guidelines on PA of at least 150–300 min of MPA per week; or at least 75–150 min of VPA per week; or an equivalent combination of MPA and VPA per week. We used the MET values 4.0 for MPA and 8.0 for VPA, according to the International Physical Activity Questionnaire (IPAQ) scoring protocol [19], and investigated the proportion of participants achieving a daily average of at least of 85.7 MET minutes running/cycling, equal to 600 MET minutes per week.

Participants who were working during the monitoring period were asked to report their daily hours at work. Average weekly work hours were calculated. The month of the first complete day of monitoring was considered the month of assessment.

### Self-reported physical activity

Information on self-reported PA was obtained using the short version of the IPAQ [20]. The IPAQ was developed for population surveillance of PA among adults and has been tested for use in the age range 15–69 years [19]. It assesses the PA categories walking, MPA, and VPA undertaken in a set of domains during the past week; 1) leisure time; 2) domestic and gardening (yard activities); 3) work; and 4) transport [19]. The IPAQ was administered twice, at examination and at the end of the monitoring period.

We used the instructions given in the IPAQ scoring protocol to calculate continuous and categorical scores [19]. The volume of activity was measured in MET minutes. The MET estimates in the IPAQ scoring protocol were derived using the Ainsworth et al. Compendium of Physical Activities [21]; walking = 3.3; moderate PA = 4; vigorous PA = 8. According to the IPAQ protocol [19] answers are considered invalid if the total time spent sitting, walking, in MPA or in VPA exceeded 1440 min, or the total time spent walking, in MPA or in VPA exceeded 960 min. One SGA and one control participant had invalid answers. One control participant did not complete the IPAQ, and 13 SGA and 17 control participants did not answer all IPAQ questions necessary to compute the three variables walking, MPA and VPA.

The IPAQ scoring protocol proposes three levels of PA: High, moderate, and low. PA is classified as high if either of the following two criteria are met: 1) ≥ three days of VPA and accumulating at least 1500 MET minutes/week; or 2) ≥ seven days of any combination of walking, MPA or VPA accumulating at least 3000 MET minutes/week. PA is classified as moderate if either of the following three criteria are met: 1) ≥ three days of VPA of at least 20 min per day; or 2) ≥ five days of MPA and/or walking of at least 30 min per day; or 3) ≥ five days of any combination of walking, MPA or VPA accumulating at least 600 MET minutes/week. Those who do not meet criteria for high or moderate PA is considered to have low PA.

### Health-related quality of life: Short Form 36 Health Survey (SF-36)

We used the Short Form 36 Health Survey (SF-36) to measure HRQoL. The SF-36 is a multi-purpose generic health questionnaire, that consists of 36 items measuring eight health domains: 1) physical functioning; 2) role limitations due to physical health problems (role-physical); 3) bodily pain; 4) general health perceptions; 5) vitality; 6) social functioning; 7) role limitations due to emotional problems (role-emotional); and 8) general mental health perceptions. The questionnaire was designed to examine health status, and the construction allows for use in research, health policy evaluations, clinical practice and general population surveys [22]. The Norwegian version of SF-36 has been evaluated in a Norwegian population of patients, and was found to have acceptable reliability and validity [23].

The SF-36 gives an insight into the individual’s understanding of their own health and provides information about well-being and ability to perform everyday tasks. The participants answer the questions by marking the option that suits them best. Raw item scores are coded, summed and transformed into an aggregate score for each of the eight domains, ranging from 0 to 100% [24, 25]. Higher scores indicate higher level of functioning and favorable health outcomes. The eight domains are aggregated into two summary measures, the physical component summary, and the mental component summary, which were used to examine associations between PA and HRQoL. The component summaries are given as T-scores, based on an average of 50 points and a standard deviation (SD) of 10 points. The domains physical functioning, role-physical and bodily pain contribute mainly to the physical component summary, while the domains social functioning, role-emotional and mental health contribute mainly to the mental component summary. The domains vitality, general health and social functioning correlate with both component summaries [25]. One SGA and one control participant only partly answered the SF-36.

### Ethical approval and consent

The Regional Committee for Medical and Health Research Ethics in Central Norway approved the study (23879). All participants gave written informed consent to participate in the project. Participants were given feedback on the examinations, and if necessary, referred to appropriate health services. Participants were offered a compensation of NOK 500 (about 50 Euros) in addition to coverage of travel expenses.

### Statistical analyses

Background characteristics of the participants born SGA and control participants were compared using Student’s t-test for continuous data, Exact Mann–Whitney U test for ordinal data and Pearson’s Chi square test for dichotomous variables.

Group differences in objectively measured daily MET minutes in the four PA categories (sedentary, on feet, on the move, running/cycling) were analyzed using linear regression, adjusted for sex, which could potentially affect PA [26]. Group differences in proportions of individuals meeting the cut-offs derived from the WHO guidelines were analyzed using Pearson’s Chi square test. Group differences in self-reported daily MET minutes of PA were analyzed using linear regression, adjusted for sex. Correlations between objectively measured and self-reported PA were analyzed using Spearman’s rho (r_s_). Group differences in self-reported PA levels were analyzed using Pearson’s Chi square test. For associations with HRQoL, daily average MET minutes in each PA category were entered separately as dependent variables, whereas group, sex, SF-36 variables, and the interaction term ‘group x SF-36 variables’ were entered as independent variables. The interaction term was added to test if the associations between PA and SF-36 were different in SGA and control participants. To reduce the number of statistical tests, we only included the summary measures of SF-36. Normality of residuals was judged by visual inspection of Q-Q plots. Due to some deviations from normality, we used bootstrapping with B = 2000 bootstrap samples and bias corrected and accelerated (BC_a_) method. A 95% confidence interval (CI) is reported where relevant, and a two-sided *p*-value < 0.05 was considered statistically significant.

STATA/MP 17.0 was used for cleaning of objectively measured PA data and SPSS 27.0 was used for data analyses.

A priori power calculations based on previous follow-up numbers in the SGA (*n* = 64) and control group (*n *= 81) suggested that we would have the power to detect differences of 0.48 SD units with an alpha-level of 0.05 and a power of 80%, and 0.67 SD units with an alpha-level of 0.01 and desired power of 90% [27].

### Supplementary analyses

We performed additional analyses to assess the robustness of the results. Objectively measured daily MET minutes in PA categories were adjusted for month of assessment and for weekly hours at work, as these factors may potentially affect the amount and type of PA [28, 29]. We conducted separate analyses for weekdays and for weekend, and we investigated the effect of a stricter requirement of at least three weekdays and one weekend day of monitoring to be included in the analyses. Furthermore, we used different MET values for walking because they vary according to the intensity of the activity. Additionally, while objective measures capture all types of walking, the IPAQ only assesses walking performed for at least 10 min at a time. We therefore examined the effect of using the MET value 3.3 from the IPAQ protocol on the objectively measured walking, instead of 2.8. We also carried out analyses of self-reported PA including participants who had only partly completed the IPAQ.

## Results

### Background characteristics

Table 1 presents background characteristics of the adults in the SGA and control group who participated at the 32-year follow-up by answering questionnaires or by wearing accelerometers. By design, the SGA group had lower weight, length, head circumference and ponderal index at birth than the control group. The mothers of the participants born SGA were 2.4 years (95% CI: 1.1 to 3.8) younger at delivery. At 32 years, participants in the SGA group were 5.0 cm (95% CI: 1.5 to 8.4) shorter than participants in the control group, otherwise there were no significant group differences in weight, BMI, parental SES, educational attainment, or age.

**Table 1:** Background characteristics of SGA and non-SGA control participants

	SGA (*n* = 56)		Control (*n* = 68)		
	Mean	(SD)	Mean	(SD)	*p*-value
Gestational age (weeks)	39.7	(1.2)	39.8	(1.2)	0.45
Birth weight (g)	2916	(205)	3695	(459)	< 0.001
Birth length (cm)^a^	48.5	(1.9)	51.1	(1.9)	< 0.001
Birth head circumference (cm)^b^	33.9	(1.1)	35.4	(1.2)	< 0.001
Ponderal index (g/cm^3^)^c^	2.6	(0.2)	2.8	(0.3)	< 0.001
Maternal age at delivery (years)^d^	28.2	(3.2)	30.7	(4.3)	< 0.001
Parental SES (1–5)^e^	3.5	(1.2)	3.7	(1.1)	0.44
Age at follow-up (years)	32.5	(0.6)	32.6	(0.5)	0.49
Height (cm)	169.5	(9.2)	174.5	(10.0)	0.005
Weight (kg)	72.6	(17.0)	76.1	(15.4)	0.23
BMI (kg/m^2^)	25.1	(4.9)	24.9	(4.3)	0.82
	n	(%)	n	(%)	
Female	31	(55)	39	(57)	0.82
Education at follow-up					
Lower secondary or lower (ISCED 1–2)	2	(4)	0	(0)	
Intermediate (ISCED 3–5)	22	(39)	23	(34)	0.24
Lower tertiary or higher (ISCED 6–8)	32	(57)	45	(66)	

*P*-values for differences based on Student’s t-test for continuous data, Exact Mann–Whitney U test for ordinal data and Pearson’s Chi square test for dichotomous variables

*ISCED* International Standard Classification of Education, *SD* standard deviation, *SES* socioeconomic status (1–5, where 5 is highest), *SGA* small for gestational age

^a^Data missing for seven SGA participants and three control participants

^b^Data missing for six SGA participants and four control participants

^c^Data missing for seven SGA participants and four control participants

^d^Data missing for six SGA participants and two control participants

^e^Data missing for nine SGA participants and eleven control participants

### Objectively measured physical activity

**Fig. 2:**
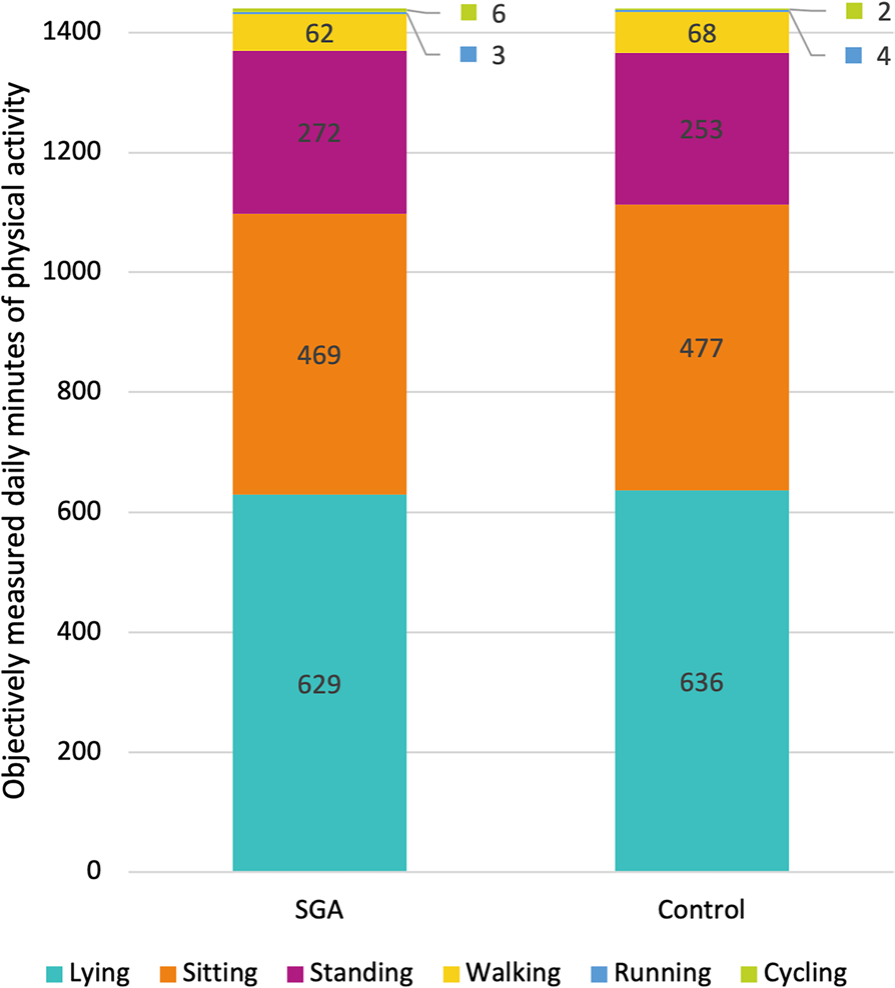
Objectively measured daily minutes of PA in SGA and non-SGA control participants. SGA = small for gestational age

The accelerometers were worn for a mean of 5.7 (SD 0.69) days in the SGA group and 5.7 (SD 0.63) days in the control group. Figure 2 presents the average daily minutes during the whole monitoring period of lying, sitting, standing, walking, running, and cycling.

Table 2 presents the average daily MET minutes in the four PA categories: sedentary (lying, sitting), on feet (standing, walking, running, cycling), on the move (walking, running, cycling) and running/cycling. The average daily MET minutes in each category did not differ between participants born SGA and control participants. The proportion of participants achieving a weekly average of at least 600 MET minutes of running or cycling was 13.5% (*n* = 5) in the SGA group and 14.0% (*n* = 6) in the control group (*p* = 0.955).

**Table 2:** Objectively measured daily MET minutes in SGA and non-SGA control participants

	SGA (*n* = 37)		Control (*n* = 43)				
	Mean	(SD)	Mean	(SD)	Mean difference (95% CI)^a^		*p*-value
Sedentary	1238	(101)	1256	(130)	-18	(-66 to 32)	0.50
On feet	708	(199)	682	(239)	25	(-73 to 123)	0.60
On the move	218	(127)	227	(113)	-9	(-61 to 51)	0.75
Running/cycling	46	(112)	37	(76)	10	(-28 to 54)	0.67

*CI* confidence interval, *SD* standard deviation, *SGA* small for gestational age

^a^Mean difference adjusted for sex. CIs and *p*-values are based on bias-corrected and accelerated bootstrap

Supplementary analyses showed no group differences in objectively measured daily MET minutes in PA categories when adjusted for month of assessment (Table A1) or for weekly hours at work (Table A2). There were no group differences in separate analyses for weekdays and weekend (data not shown), or when including only participants with at least three weekdays and one weekend day of monitoring (data not shown). Using different MET values for walking had negligible influence on the estimated mean difference in daily MET minutes on feet and on the move.

### Self-reported physical activity

**Fig. 3:**
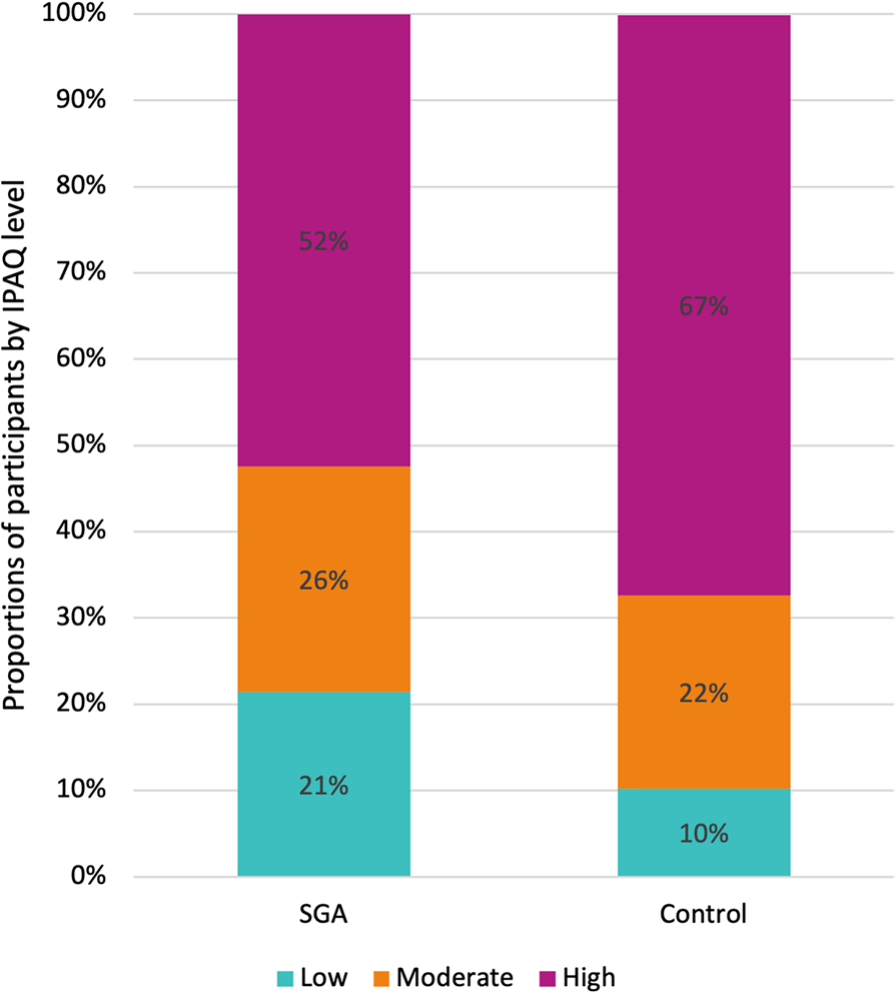
Proportions of low, moderate, and high IPAQ levels in SGA and non-SGA control participants. IPAQ = International Physical Activity Questionnaire; SGA = small for gestational age

Table 3 presents average daily MET minutes of walking, MPA and VPA obtained from the first administration of the IPAQ. Average daily MET minutes in each PA category did not differ between participants born SGA at term and control participants. There were no group differences in proportions of low, moderate, and high IPAQ levels (Fig. 3) and no group differences in self-reported PA after seven days of monitoring (Table A3). There were no group differences when we included participants who had only partly competed the IPAQ (data not shown).

**Table 3:** Self-reported daily MET minutes of PA at examination in SGA and non-SGA control participants

	SGA (*n* = 42)		Control (*n* = 49)				
	Mean	(SD)	Mean	(SD)	Mean difference (95% CI)^a^		*p*-value
Walking	281	(367)	219	(325)	63	(-79 to 214)	0.39
Moderate physical activity	282	(377)	276	(403)	6	(-140 to 154)	0.94
Vigorous physical activity	355	(378)	428	(371)	-73	(-224 to 77)	0.35

*CI* confidence interval, *SD* standard deviation, *SGA* small for gestational age

^a^Mean difference adjusted for sex. CIs and p-values are based on bias-corrected and accelerated bootstrap

The correlations between objectively measured and self-reported PA during the monitoring period were lowest for walking (r_s_ = 0.002 in the SGA and r_s_ = 0.28 in the control group) and highest between self-reported MVPA and objectively measured running/cycling (r_s_ = 0.66 in the SGA and r_s_ = 0.41 in the control group).

### Associations with health-related quality of life

Table 4 presents the associations between objectively measured daily MET minutes spent in the four PA categories and the physical and the mental component summary of SF-36. We found no group differences in the associations between objectively measured daily MET minutes in each category and the SF-36 scores. In the SGA group, an increase of one MET minute of objectively measured time on the move was associated with an increase of 4.0 (95% CI: 0.6 to 6.5, *p* = 0.009) points in the physical component summary.

**Table 4:** Associations between objectively measured daily MET minutes and SF-36 in SGA and non-SGA control participants

		SGA (*n* = 36)			Control (*n* = 42)			SF36 x group
PA category	SF-36 scores	B	(95% CI)	*p*-value	B	(95% CI)	*p*-value	*p*-value^a^
Sedentary	Physical component summary	-2.6	(-5.7 to 2.3)	0.11	-0.6	(-8.2 to 5.3)	0.85	0.60
Sedentary	Mental component summary	0.3	(-5.6 to 5.3)	0.91	-2.3	(-7.6 to 1.2)	0.30	0.41
On feet	Physical component summary	5.5	(-4.8 to 11.2)	0.09	0.9	(-12.0 to 14.7)	0.88	0.51
On feet	Mental component summary	1.9	(-6.8 to 15.4)	0.68	4.2	(-2.7 to 14.6)	0.31	0.68
On the move	Physical component summary	4.0	(0.6 to 6.5)	0.009	-0.4	(-6.2 to 6.4)	0.87	0.25
On the move	Mental component summary	2.9	(-3.1 to 15.0)	0.46	1.7	(-1.2 to 6.1)	0.36	0.72
Running/cycling	Physical component summary	0.7	(-0.9 to 2.0)	0.37	2.1	(-0.4 to 5.2)	0.17	0.66
Running/cycling	Mental component summary	3.9	(0.8 to 12.3)	0.14	0.1	(-1.3 to 2.5)	0.94	0.14

Regression coefficient B for SF-36 scores in a linear regression with objectively measured PA categories as dependent variables and SF-36, group, and SF-36 × group (indicating between-group differences) as independent variables, adjusted for sex

*MET* metabolic equivalent of task, *SF-36* Short Form 36 Health Survey, *SGA* small for gestational age

^a^*p*-value for group differences in associations between objectively measured daily MET minutes in PA categories and SF-36 scores

Table 5 presents the associations between self-reported daily MET minutes spent in PA categories and the physical and the mental component summary of SF-36. We found no group differences in the associations between self-reported daily MET minutes in each category and the SF-36 component summaries. However, in the control group, self-reported VPA was associated with the physical and mental component summary (Table 5).

**Table 5:** Associations between self-reported daily MET minutes and SF-36 in SGA and non-SGA control participants

		SGA (*n* = 41)			Control (*n* = 49)			SF36 x group
PA category	SF-36 scores	B	(95% CI)	*p*-value	B	(95% CI)	*p*-value	*p*-value^a^
Walking	Physical component summary	2.5	(-14.5 to 12.6)	0.71	4.9	(-9.5 to 22.2)	0.58	0.83
Walking	Mental component summary	3.8	(-3.2 to 11.3)	0.36	4.4	(-5.4 to 18.4)	0.20	0.93
MPA	Physical component summary	6.1	(-5.5 to 17.3)	0.24	-17.6	(-41.3 to 1.2)	0.12	0.10
MPA	Mental component summary	-1.6	(-12.7 to 5.6)	0.72	9.6	(0.8 to 29.9)	0.07	0.18
VPA	Physical component summary	-2.6	(-33.1 to 14.3)	0.80	-16.7	(-33.7 to -4.0)	0.04	0.30
VPA	Mental component summary	4.4	(-3.6 to 10.8)	0.23	15.0	(5.3 to 32.3)	0.003	0.17

Regression coefficient B for SF-36 scores in a linear regression with self-reported PA categories as dependent variables and SF-36, group, and SF-36 × group (indicating between-group differences) as independent variables, adjusted for sex

*MET* metabolic equivalent of task, *MPA* moderate physical activity, *SF-36* Short Form 36 Health Survey, *SGA* small for gestational age, *VPA* vigorous physical activity

^a^*p*-value for group differences in associations between self-reported daily MET minutes in PA categories and SF-36 scores

## Discussion

Overall, objectively measured and self-reported PA did not differ between term-born SGA and non-SGA control participants in adulthood. We found that around 14% of the participants in each group met the PA recommendations based on objectively measured time spent running or cycling. The associations between PA and HRQoL did not differ between the groups.

Strengths of this study include the prospective design and the use of objectively measured PA. However, there are several limitations that should be considered when interpreting the results. Due to technical or practical issues, we were unable to obtain valid accelerometer data from all participants who agreed to wear them. Furthermore, the data collection was conducted partly during the COVID-19 pandemic, which prevented some participants from meeting at the clinical examination. Loss to follow-up is challenging in longitudinal studies and the relatively small sample size could reduce the power to detect differences between the groups. The findings of no difference between groups should therefore be interpreted with caution. According to the mean differences relative to the SDs in the control group, we may exclude large group differences, however, small to moderate differences (less than approximately 0.5 SD) cannot be excluded. Furthermore, our findings may have limited generalizability due to the limited sample size. However, 65% of the invited in both groups participated at the current follow-up, and we found no substantial differences between participants and those who did not consent to participate. Thus, bias due to loss to follow-up is unlikely and we can assume that the participants were representative of the initial sample.

The use of accelerometers to objectively measure PA has advantageous properties over subjective methods [30]. In particular, self-reported PA could be subject to misclassification, recall error and social desirability bias, which is avoided by objective measurements [31]. Using two tri-axial accelerometers enables accurate detection of periods with lying down, sitting, standing, walking, running, and cycling [16]. In contrast, previous studies on the association between birth weight and PA that have used accelerometers have quantified PA by counts per minute [32–35]. Although studies validating the IPAQ short form have yielded inconsistent results [36], the advantage of using self-reports was that more subjects were able to participate by answering questionnaires only, as some were unable to meet at the clinical examination. Additionally, using self-reports allowed us to discuss our findings in relation to previous literature. Due to the different aspects and dimensions studied by objective measures and self-reports, it is advised to use both methods to collect comprehensive and complementary PA data [37]. A further strength to our study was the assessment of associations between PA and the participants’ health and functioning from their own point of view. The SF-36 has been validated in more than 25 countries [38, 39], and the Norwegian version was found to have acceptable reliability and validity in a Norwegian population of patients [23].

The WHO recommend that adults should do at least 150–300 min of MPA or 75–150 min of VPA, or a combination of both per week [8]. On an absolute scale, moderate intensity refers to PA ranging from 3 to 6 METs, while vigorous intensity refers to PA above 6 METs [40]. In this study we used the MET values 4.0 for MPA and 8.0 for VPA, according to the IPAQ protocol [19], to calculate cut-offs from the guidelines. In analyses of the correlation between objectively measured and self-reported MVPA, we used objectively measured running and cycling only, leaving out walking, because when participants report MPA in the IPAQ, they are asked to not include walking. Furthermore, accelerometers measure all intensities of walking throughout the day.

To calculate daily MET minutes on the move (walking, running, and cycling), we used the MET value of 2.8 for walking, considered appropriate for accelerometer-measured activity. However, the IPAQ proposes a MET value of 3.3 for walking, as participants only report walking performed for at least 10 min at a time. We used the MET value from the IPAQ scoring protocol to assess objectively measured MET minutes on feet (standing, walking, running, and cycling) and on the move (walking, running, and cycling). There were no group differences in objectively measured daily MET minutes on feet or on the move using either MET value.

In this study, we found no difference in objectively measured PA between participants born SGA at term and control participants. While we are not aware of other studies assessing PA in term-born SGA adults, some studies have examined associations between birth weight and objectively measured PA or sedentary time. In children and adolescents, Ridgway et al. [32] found no association between birth weight and total PA or sedentary time in a combined analysis of four studies (*n* = 4170) of uniaxial accelerometry-measured PA. Also, Kehoe et al. [33] and Mattocks et al. [34] found no associations between birth weight and objectively measured PA in 663 Indian children aged 6–10 years and 5451 UK children aged 11–12 years who wore Actigraph accelerometers for seven days. On the contrary, Hildebrand et al. [35] reported that a higher birth weight was associated with more sedentary time measured by a waist worn accelerometer in 6–18-year-old individuals. However, the associations seemed to be driven mainly by the extreme birth weight categories (< 2.75 and > 4.75 kg).

In the present study, we found no difference in self-reported PA between participants born SGA at term and control participants. In a meta-analysis by Andersen et al. [41], birth weight showed a reverse U-shaped association with self-reported leisure-time PA in adolescents and adults. However, this association was negligible within normal birth weights. Moreover, Hallal et al. [42] studied 4453 adolescents aged 10–12 years and found no association between birth weight and sedentary lifestyle. On the other hand, Fernandes et al. [43] and Elhakeem et al. [44] reported that birth weight at or below 2500 g was a risk factor for sedentary behavior measured by the IPAQ in 2063 Brazilian young adults (23–25 years) and less leisure time PA across adulthood in British singletons born in 1946, respectively. However, the birth weight cut-offs used in the studies of Fernandes et al. [43] and Elhakeem et al. [44] were lower than the mean birth weight of the SGA group in our study which may explain some of the discrepancy. Additionally, the participants in the British study were followed up across adulthood from 36 to 68 years, and as PA declines with age [45], this may contribute to the inconsistent results.

A systematic review reported that the correlation between the IPAQ and objective measures overall was lower than the acceptable standard of 0.50, and that the IPAQ overestimated PA in most studies [36]. However, for walking and MVPA, the correlations were acceptable in some studies [36]. In contrast, we found low correlations for walking, but acceptable correlation for MVPA in the SGA group, even though both groups seemed to overestimate their MVPA by self-report.

Although PA has been associated with HRQoL in the general population [10, 11], there are no studies of the association between objectively measured PA and HRQoL in adults born SGA at term. In the present study, we found no group differences in the associations between objectively measured or self-reported PA and HRQoL. In the SGA group, objectively measured time on the move was positively associated with HRQoL. This may be plausible as the SGA group reported more problems with daily activities due to their physical health at 32 years [12]. The physical component of SF-36 is a summary of items concerning performance of activities, such as running, lifting, domestic life, walking distances and activities of daily living (physical functioning), limitations due to physical health and bodily pain, which seems highly correlated to time on the move.

This study is the first to investigate objectively measured PA among adults born SGA at term. It is reassuring that we found no differences in objectively measured or self-reported PA between participants born SGA at term and control participants. However, only 14% of the participants in both groups met the cut-offs derived from WHO guidelines on PA based on objectively measured daily MET minutes running or cycling. The increased risk of poor physical and mental health in those born SGA, could be mitigated through lifestyle changes, such as implementing more PA into their daily life [46].

## Conclusions

Overall, we found no differences in objectively measured and self-reported PA or associations with HRQoL between term-born SGA and non-SGA control participants in adulthood.

### Supplementary Information

**Additional file 1: Table A1**. Objectively measured daily MET minutes in SGA and non-SGA control participants, adjusted for sex and month.

**Additional file 2: Table A2. **Objectively measured daily MET minutes in SGA and non-SGA control participants, adjusted for sex and work hours.

**Additional file 3: Table A3. **Self-reported daily MET minutes of PA in SGA and non-SGA control participants after the monitoring period.

## Data Availability

The datasets generated and/or analyzed during the current study are not publicly available because permission has not been applied for from neither the participants nor the Ethical Committee but are available from the corresponding author on reasonable request.

## Abbreviations

BCa: Bias-corrected and accelerated bootstrap
BMI: Body mass index
CI: Confidence interval
HRQoL: Health-related quality of life
IPAQ: International Physical Activity Questionnaire
ISCED: International Standard Classification of Education
LBW: Low birth weight
LMP: Last menstrual period
MPA: Moderate physical activity
MET: Metabolic equivalent of task
PA: Physical activity
SD: Standard deviation
SES: Socioeconomic status
SF-36: Short Form 36 Health Survey
SGA: Small for gestational age
VPA: Vigorous physical activity
