# Supplementary material for: Physical activity and associations with health-related quality of life in adults born small for gestational age at term: a prospective cohort study
Source: BMC Pediatr. 2023 Aug 28;23:430. doi: 10.1186/s12887-023-04256-y (PMC10464269; doi:10.1186/s12887-023-04256-y)
Supplement: Supplementary file 1 — Additional file 1: Table A1. Objectively measured daily MET minutes in SGA and non-SGA control participants, adjusted for sex and month. [file 12887_2023_4256_MOESM1_ESM.docx]

**Table A1.** Objectively measured daily MET minutes in SGA and non-SGA control participants, adjusted for sex and month.

|  | SGA (n = 37) | |  | Control (n = 43) | |  |  |  |  |  | |  |
| --- | --- | --- | --- | --- | --- | --- | --- | --- | --- | --- | --- | --- |
|  | Unadjusted mean | (SD) |  | Unadjusted mean | (SD) | Mean difference adjusted for sex (95% CI) | | p-value |  | Mean difference adjusted for sex and month of monitoring (95% CI) | | p-value |
| Sedentary | 1238 | (101) |  | 1256 | (130) | -18 | (-66 to 32) | 0.50 |  | -14 | (-63 to 37) | 0.62 |
| On feet | 708 | (199) |  | 682 | (239) | 25 | (-73 to 123) | 0.60 |  | 23 | (-79 to 122) | 0.65 |
| On the move | 218 | (127) |  | 227 | (113) | -9 | (-61 to 51) | 0.75 |  | -2 | (-53 to 55) | 0.94 |
| Running/cycling | 46 | (112) |  | 37 | (76) | 10 | (-28 to 54) | 0.67 |  | 17 | (-21 to 62) | 0.51 |

Confidence intervals and p-values based on bias-corrected and accelerated bootstrap (BCa).

CI = confidence interval; MET = metabolic equivalent of task; SD = standard deviation; SGA = small for gestational age.
