# Supplementary material for: Physical activity and associations with health-related quality of life in adults born small for gestational age at term: a prospective cohort study
Source: BMC Pediatr. 2023 Aug 28;23:430. doi: 10.1186/s12887-023-04256-y (PMC10464269; doi:10.1186/s12887-023-04256-y)
Supplement: Supplementary file 2 — Additional file 2: Table A2. Objectively measured daily MET minutes in SGA and non-SGA control participants, adjusted for sex and work hours. [file 12887_2023_4256_MOESM2_ESM.docx]

**Table A2.** Objectively measured daily MET minutes in SGA and non-SGA control participants, adjusted for sex and work hours.

|  | SGA (n = 29) | |  | Control (n = 27) | |  | |  |  |  | |  |
| --- | --- | --- | --- | --- | --- | --- | --- | --- | --- | --- | --- | --- |
|  | Unadjusted mean | (SD) |  | Unadjusted mean | (SD) | Mean difference adjusted for sex (95% CI) | | p-value |  | Mean difference adjusted for sex and hours at work (95% CI) | | p-value |
| Sedentary | 1237 | (104) |  | 1238 | (123) | -2 | (-67 to 68) | 0.95 |  | -19 | (-84 to 59) | 0.54 |
| On feet | 713 | (200) |  | 735 | (195) | -20 | (-128 to 88) | 0.72 |  | 17 | (-94 to 119) | 0.76 |
| On the move | 223 | (135) |  | 237 | (104) | -15 | (-78 to 54) | 0.67 |  | -2 | (-81 to 78) | 0.96 |
| Running/cycling | 51 | (124) |  | 42 | (88) | 6 | (-46 to 72) | 0.86 |  | 17 | (-50 to 100) | 0.72 |

Confidence intervals and p-values based on bias-corrected and accelerated bootstrap (BCa).

CI = confidence interval; MET = metabolic equivalent of task; SD = standard deviation; SGA = small for gestational age.
