# Supplementary material for: Physical activity and associations with health-related quality of life in adults born small for gestational age at term: a prospective cohort study
Source: BMC Pediatr. 2023 Aug 28;23:430. doi: 10.1186/s12887-023-04256-y (PMC10464269; doi:10.1186/s12887-023-04256-y)
Supplement: Supplementary file 3 — Additional file 3: Table A3. Self-reported daily MET minutes of PA in SGA and non-SGA control participants after the monitoring period. [file 12887_2023_4256_MOESM3_ESM.docx]

**Table A3.** Self-reported daily MET minutes of PA in SGA and non-SGA control participants after the monitoring period.

|  | SGA (n = 25) | |  | Control (n = 36) | |  |  |  |
| --- | --- | --- | --- | --- | --- | --- | --- | --- |
|  | Mean | (SD) |  | Mean | (SD) | Mean difference (95% CI)^a^ | | p-value |
| Walking | 215 | (265) |  | 257 | (378) | -43 | (-224 to 124) | 0.62 |
| Moderate physical activity | 170 | (305) |  | 206 | (257) | -35 | (-173 to 108) | 0.64 |
| Vigorous physical activity | 296 | (340) |  | 272 | (277) | 24 | (-130 to 194) | 0.78 |

CI = confidence interval; MET = metabolic equivalent of task; SD = standard deviation; SGA = small for gestational age.

^a^Mean difference adjusted for sex, confidence interval and p-value based on bias-corrected and accelerated bootstrap (BCa).
